# 

**Published:** 1980

**Authors:** 


JANUARY/APRIL 1980
VOLUME 95 (i/ii)
NUMBER 353/354
Published Quarterly
Annual Subscription £6 Post Free

### BRISTOL MEDICO-CHIRURGICAL JOURNAL

### Contents

3 **The Medical Adviser and General Practitioner Trials**

R. P. Bax

7 **Dermatological do-it-yourself**
J. L. Burton

9 **Imitation**
Philip Radford

11 **Abstracts**

Established 1883

### BRISTOL MEDICO-CHIRURGICAL JOURNAL

Incorporating the Medical Journal of the South West

### Editorial Staff

EDITOR
*Dr. D. S. Reeves*
*Southmead Hospital, Bristol, BS10 5NB*
ASSISTANT EDITOR
*Dr. A. P. Radford*
MEMBERS
*The Hon. Treasurer of the Society*
*The Hon. Secretary of the Society*
SECRETARY
*Mr. B. P. Jones, The Library, Medical School, University Walk, Bristol, BS8 1TD*

### Bristol Medico-Chirurgical Society

PRESIDENT 1979/80
*Dr. D. S. Ractliffe*
PRESIDENT ELECT
*Dr. N. J. Brown*
HONORARY SECRETARY
*A. J. Webb, Esq., 7 Percival Road, Bristol, BS8 3LE*
HONORARY TREASURER
*Dr. J. Penry, Southmead Hospital, Bristol, BS10 5NB*

The Society was founded in 1874. In 1883 publication of the Journal began and has continued ever since then. During the years 1949 to 1962 it appeared under the title of 'The Medical Journal of the South West'.

### Notice to Contributors

*The Journal is especially concerned to provide a place for the recording of medical thought, interests, and practice in and around Bristol. It therefore welcomes articles that, as well as being of immediate interest to members, will document the local and contemporary medical scene for our successors.*

*Original articles are invited provided that they have not been published elsewhere. Illustrations should be supplied ready for reproduction. Line drawings should be in Indian ink on firm white paper or board. The author will be informed if it is found necessary to ask him to pay for the printing of photographs etc.*

*The following contributions will be especially welcome; notes of forthcoming events, meetings held, degrees conferred, staff appointments, honours and public appointments and other local news.*

*Advice on preparation can be obtained from the Editor.*

Printed by the University of Bristol Printing Unit

### University of Bristol

### FACULTY OF MEDICINE

### The Degree of Doctor of Medicine

**January 1980**

Beverley John BURKE
Angela Mary SHEPHERD
Richard WINDLE

**March 1980**

Graham John DAVIES

### The Degree of Doctor of Philosophy

**January 1980**

Mark Anthony BISHOP
Katrina Jane DILLON
Peter Michael HUYTON
Malcolm Anthony MOORE
Kenton Lloyd MORGAN
Reginald Anthony NICHOLLS

### The Degree of Master of Science in Medical Sciences

**January 1980**

Abolhassan FAJRI
Encio Kofi KPEDEKPO
Laith Abdul Jalil JAWAD

**March 1980**

Vassilis NATSIKOS

### The Degree of Master of Dental Surgery

**March 1980**

Ernest Siew Whye WONG

### A SELECTION OF RECENT ADDITIONS TO THE MEDICAL LIBRARY

*Continued from:* **July/October 1979**

ISAACS, A. D. & POST, F., Studies in geriatric psychiatry. (Wiley)

JEFFCOATE, S. L. & HUTCHINSON, J. S. M., The endocrine hypothalamus. (Acad. Pr.)

JELJASZEWICZ, J. & WADSTROM, T., Bacterial toxins and cell membranes. (Acad. Pr.)

JERESATY, R. M., Mitral valve prolapse. (Raven Pr.)

JOHNSON, P. C., Peripheral circulation. (Wiley)

JONES, R. E., The vertebrate ovary. (Plenum Pr.)

JONES, R. V. H., Running a practice. (Croom Helm)

KOCHETKOVA, V. I., Paleoneurology. (Halsted Press)

KOPROWSKI, H., Neoplastic transformation: mechanisms and consequences. (Dahlem Konferenzen)

KREBS, B. P., et al.: Clinical application of carcinoembryonic antigen assay. (Excerpta Medica)

KREUTNER, A. K. & HOLLINGSWORTH, D. R., Adolescent obstetrics and gynecology. (Year Book Med. Publ.)

LAMB, D. R., Physiology of exercise. (Macmillan)

LEDLEY, R. S., et al.: Cross-sectional anatomy: an atlas for computerized tomography. (Williams & Wilkins)

LILLINGTON, G. A. & JAMPLIS, R. W., A diagnostic approach to chest diseases. (Williams & Wilkins)

MACDONALD, A. G. & WANN, K. T., Physiological aspects of anaesthetics and inert gases. (Acad. Pr.)

MATTER, P., The open fracture: assessment, surgical treatment, and results. (Huber)

MIDGLEY, A. R. & SADLER, W. A., Ovarian follicular development and function. (Raven Pr.)

MOE, J. H., et al.: Scoliosis and other spinal deformities. (Saunders)

MORELL, P., Myelin. (Plenum Pr.)

MOTTA, P., et al.: The liver: an atlas of scanning electron microscopy. (Igaku-Shoin)

OPFERKUCH, W., et al.: Clinical aspects of the complement system. (Thieme)

OXFORD GP TRAINEE GROUP, A guide to general practice. (Blackwell)

PAINE, L. H. W., Health care in big cities. (Croom Helm)

PATTYN, S. R., Ebola virus haemorrhagic fever. (Elsevier)

PAYKEL, E. S. & COPPEN, A., Psychopharmacology of affective disorders. (Clarendon Press)

PIA, H. W. & DJINDJIAN, R., Spinal angiomas: advances in diagnosis and therapy. (Springer)

PRITCHARD, P., Manual of primary health care: its nature and organization. (Oxford Univ. Pr.)

RAINER, H., Immunotherapy of malignant diseases. (Schattauer)

REID, D. A. C. & GOSSET, J., Mutilating injuries of the hand. (Churchill Livingstone)

REMMER, H., et al.: Primary liver tumors. (MTP)

RICCI, M., et al.: Developments in clinical immunology. (Acad. Pr.)

ROSE, F. C., Clinical neuroimmunology. (Blackwell)

ROTTENBERG, D. A. & HOCHBERG, F. H., Neurological classics in modern translation. (Hafner Press)

RUSSEL, D. H. & DURIE, B. G. M., Polyamines as biochemical markers of normal and malignant growth. (Raven Pr.)

SAIDMAN, L. J. & SMITH, N. T., Monitoring in anesthesia. (Wiley)

SELIKOFF, I. J. & LEE, D. H. K., Asbestos and disease. (Acad. Pr.)

SHARMA, R. K. & CRISS, W. E., Endocrine control in neoplasia. (Raven Pr.)

SHERLOCK, S. & SUMMERFIELD, J. A., A colour atlas of liver disease. (Wolfe)

SILVERBERG, S. G., Surgical pathology of the uterus. (Wiley)

SLATER, T. F., Biochemical mechanisms of liver injury. (Acad. Pr.)

SLEISENGER, M. H. & FORDTRAN, J. S., Gastrointestinal disease: pathophysiology, diagnosis, management. (Saunders) 2 vols.

SMITH, S., The maltreatment of children: a comprehensive guide to the battered baby syndrome. (MTP)

STEPHEN, W. J., An analysis of primary medical care: an international study. (Cambridge Univ. Pr.)

STILLMAN, R. C. & WILLETTE, R. E., The psychopharmacology of hallucinogens. (Pergamon Pr.)

TAGNON, H. J. & STAQUET, M. J., Recent advances in cancer treatment. (Raven Pr.)

TAYMOR, M. L., Infertility. (Grune & Stratton)

THOMAS, P., Pharmacology of the eye. (Lloyd-Luke)

THOMPSON, R. H. & GREEN, J. R., Neoplasia in the central nervous system. (Raven Pr.)

TOVELL, H. M. M. & DANK, L. D., Gynecologic operations. (Harper & Row)

VRBOVA, G., et al.: Nerve-muscle interaction. (Chapman & Hall)

WACHTEL, P. L., Psychoanalysis and behaviour therapy. (Basic Books)

WAGENVOORT, C. A. & WAGENVOORT, N., Pathology of pulmonary hypertension. (Wiley)

WARREN, H. S. & MAHOUD, A. A. F., Geographic medicine for the practitioner — algorithms in the diagnosis and management of exotic diseases. (Chicago Univ. Pr.)

WATERSON, A. P. & WILKINSON, L., An introduction to the history of virology. (Cambridge Univ. Pr.)

WEISS, E. B., Status asthmaticus. (Univ. Park Pr.)

WEST, D. J., et al.: Understanding sexual attacks. (Heinemann)

WHEATLEY, D., Stress and the heart. (Raven Pr.)

YEN, S. S. C. & JAFFE, R. B., Reproductive endocrinology: physiology, pathophysiology, and clinical management. (Saunders)

YOUNG, J. A. & VAN LENNEP, E. W., The morphology of salivary glands. (Acad. Pr.)

